# Crossmodal correspondences and interactions between texture and taste perception

**DOI:** 10.1177/20416695231163473

**Published:** 2023-03-30

**Authors:** Eleftheria Pistolas, Johan Wagemans

**Affiliations:** Laboratory of Experimental Psychology, Department of Brain and Cognition, University of Leuven, Belgium

**Keywords:** taste, texture, mouthfeel, crossmodal correspondences, multisensory perception

## Abstract

In recent years, awareness of the influence of different modalities on taste perception has grown. Although previous research in crossmodal taste perception has touched upon the bipolar distinction between softness/smoothness and roughness/angularity, ambiguity largely remains surrounding other crossmodal correspondences between taste and other specific textures we regularly use to describe our food, such as crispy or crunchy. Sweetness has previously been found to be associated with soft textures but our current understanding does not exceed the basic distinction made between roughness and smoothness. Specifically, the role of texture in taste perception remains relatively understudied. The current study consisted of two parts. First, because of the lack of clarity concerning specific associations between basic tastes and textures, an online questionnaire served to assess whether consistent associations between texture words and taste words exist and how these arise intuitively. The second part consisted of a taste experiment with factorial combinations of four tastes and four textures. The results of the questionnaire study showed that consistent associations are made between soft and sweet and between crispy and salty at the conceptual level. The results of the taste experiment largely showed evidence in support of these findings at the perceptual level. In addition, the experiment allowed for a closer look into the complexity found regarding the association between sour and crunchy, and bitter and sandy.

The consumption of food has become an elaborate gastronomic experience on many occasions. Think of the exclusive sushi restaurants that host one company each evening, during which the sushi chef elaborately engages with the clients seated in front of his working space and presents one piece of sushi at a time, giving the clients the time to appreciate each piece individually. In recent years, more awareness has grown of the influence of different modalities on taste perception and the way in which different modalities add to the gastronomic experience. For instance, Woods and Spence (2016) have found consistent color and taste associations. An aspect that is relatively understudied is how texture contributes to taste perception.

## Texture and Taste Associations

Although previous research has addressed the association between basic tastes and roughness or smoothness (Cheskin, 1967; Dichter, 1971; Spence & Ngo, 2012), our current understanding does not exceed this basic distinction. The lack of solid empirical findings regarding these specific texture–taste associations raises the question whether such consistent associations exist altogether. Izutsu and Wani (1985) suggested that texture is the most important sensory property in foods with relatively low flavor intensity and expressed the importance of more quantitative texture studies as well as research on the traditional four or five basic tastes. Previous studies that draw attention to the prominence of texture in food acceptance and food consumption in general suggest texture to be an aspect of taste perception with considerable value. Werthmann and colleagues (2015) have found that sensory features in texture affect the willingness to try novel foods. In addition, the results of this study convey that alterations in the texture of food (i.e., baseline yogurt, yogurt with little pieces, and yogurt with bigger pieces) influence the consumption and acceptance of food. In light of these findings, it is evident that the role of texture in taste perception needs to be studied further.

Our current understanding regarding texture and taste associations is rather limited. Spence and Ngo (2012) argued that round shapes and textures are more often associated with sweetness, whereas angular shapes and textures are more often associated with bitterness. These associations seem to be anticipated by the food industry through label designs, etc. Angular shapes can be found more often on labels of carbonated water bottles than on labels of still water bottles (Spence & Ngo, 2012). Packaging color has also been found to influence expected flavor, texture, and liking (Baptista et al., 2021). In terms of the texture of the food product itself, creaminess is considered to carry a hedonic aspect (Frøst & Janhøj, 2007), often associated with “pleasantness” (Antmann et al., 2010). In addition, the smoothness/roughness of a food product has previously been found to influence taste perception (Slocombe et al., 2016). As such, food with a rough surface was rated more sour than food with a smooth surface. The importance of studying these interactions more closely is further supported by the finding that even a small amount of juice that was released from a tissue had the ability to trigger the mechanisms of sweetness perception in a study that investigated the relationship between juiciness and sweetness of fruit (Harker et al., 2006).

## Limitations of the Predominant Type of Investigation of Food Texture

The current literature on food texture mainly focuses on chemical considerations (Thun et al., 2022; Wagoner et al., 2019; Wilailux et al., 2019), in particular on the way in which food is broken down in the mouth, on how this influences the flavor sensors as the chemical compounds and food structures transform (Tournier et al., 2007), and on how this influences the perceived texture. In terms of physicochemical mechanisms, research in this field has mainly focused on the role of mastication, specifically aspects such as mechanical deformation, changes in temperature, dilution, and enzymatic breakdown due to saliva (Linforth & Taylor, 2006). Such research does not directly address the issue of taste–texture associations and does not contribute to clarifying the persisting taste–texture ambiguity. As opposed to varying one particular texture and measuring how this variation influences the extent to which the taste is perceived, in the present study, we are interested in learning how several textures influence the perception of basic tastes and how certain combinations of texture and taste are perceived compared to others.

## Cross-Cultural Differences in the Attention for Mouthfeel

Regarding the perception of texture attributes, an underlying factor that previously has been suggested to affect taste is the bodily sensation or “mouthfeel” (Galmarini et al., 2013), defined as the perception of texture through tactile properties during food consumption (Stokes et al., 2013). The attention for mouthfeel in cuisine differs cross-culturally. The Japanese language, for instance, contains 408 expressions to describe the sensation in the mouth when consuming food, while only 78 expressions describe mouthfeel in the American English language (Drake, 1989). A prominent example of a food product that is widely used in Japanese cuisine to add certain textures to a dish is seaweed, which can be used to add a crunch, create a crisp or even generate a level of chewiness to a dish. The appeal of seaweed lies partly in its potential to be manipulated into the desired texture and consequently creating an elevated gastronomic experience due to the complimenting of textures to tastes, anticipating and responding to the sensory influence of mouthfeel on taste perception and appreciation. Another part of the appeal, however, is the taste, given that seaweed is inherently intertwined with the discovery of the taste “umami.” The Japanese chemist Kikunae Ikeda discovered the fifth basic taste while studying Japanese soups, in particular “dashi,” a soup broth that consists of “konbu,” a seaweed. Ikeda attributed the pleasant taste of Japanese soups to the presence of a considerable concentration of monosodium glutamate in konbu (Mouritsen, 2017). The term “umami” was proposed by Ikeda to describe the taste that emerges from the presence of a considerable concentration of monosodium glutamate in konbu (Ikeda, 2002).

Returning to the linguistic difference between Japan and the United States of America concerning mouthfeel, the attention for this component of the gastronomic experience does not seem as deeply rooted in Western culture. Aside from the attention for mouthfeel, the common use of seaweed in dishes is not as established and appreciated in the West as it is the Far East. Regardless of whether this cross-cultural difference may be due to cross-cultural differences in eating habits, preference or other factors, it is important to find innovative ways to use and transform tactile food properties based on results that demonstrate which combinations make food more tasteful. Despite the looming consequences of human demand on natural capital, and given that macro- and microalgae form the largest biomass on this planet (Mouritsen, 2017), people will not adhere to appeals for more sustainable food options unless they like the taste and mouthfeel of the alternatives. These efforts are not uniquely important for a wider spread of seaweed consumption, although we use seaweed to illustrate cross-cultural differences in the attention dedicated to the mouthfeel component of the gastronomic experience. It remains relevant to learn how to prepare alternative food products that are naturally present in abundance on earth, in response to what speaks to the senses cross-culturally. A first step toward a better understanding of the value and potential of texture in the gastronomic experience requires an investigation of the role of texture in taste perception.

## The Current Study

The current study aims, in the first place, to investigate whether consistent associations between texture and taste exist at all. Considering the importance of environment and atmosphere in the gastronomic experience, the experiment was conducted in natural settings (Spence & Piqueras-Fiszman, 2014).

Participants were provided with edible samples of varying texture or taste. The experiment itself consisted of sampling the stimuli while following online instructions and answering questions. The online instructions and questions enabled participants to take part in this study from the comfort and safety of their home/student residence (which was crucial for the feasibility of the study during the covid-pandemic).

In view of the previously identified gap between the physicochemical literature and our main focus, the present study was largely exploratory. The main research question of the taste experiment was how texture and taste associations arise by means of the congruent and incongruent pairing of these two variables and whether congruency affects basic taste perception. Labrecque et al. (2013) proposed that some effects of color, in research regarding color and taste correspondences, also emerge from acquired knowledge. This acquired knowledge is particularly important with respect to the congruency between color and product attributes. For instance, a color that is incongruent with the person's perception of the product has previously been found to influence smell (Zellner et al., 1991), taste (Koch & Koch, 2003) and flavor identification (Garber et al., 2000). In order to gather more insight in the possible reinforcement of taste by pairing it with a congruent texture, a short questionnaire aimed to create the basis to form congruent and incongruent pairs for the taste experiment. The question that provisionally remains is whether consistent associations between taste and texture exist and therefore whether we can speak of congruent pairings to begin with. Our questionnaire study will not be able to pinpoint the specific source of such congruencies (e.g., linguistic, semantic, affective, innate, or acquired through associative learning; see Spence, 2011), if they exist at all. In case the short questionnaire would not indicate the presence of congruency, the data analysis of the taste experiment would focus on investigating the influence of texture on the extent to which a certain taste is perceived for each texture.

As an effort to consider the possibility of experience, or acquired knowledge, acting as a confounding variable regarding crossmodal associations, the influence of experience on texture and taste associations was incorporated in this investigation as well. Similarly, cross-cultural differences may exist with acquired knowledge and habits ultimately underlying texture and taste associations. The influence of culture on texture and taste associations was therefore also studied by including a few relevant demographic questions. Another aspect that may be of importance and that was therefore incorporated into the study is liking. Finally, because of the aforementioned relevance of mouthfeel, the present study also assessed bodily sensation and its potential role in the association between texture and taste. As a result, our present study has assessed several issues, although we try to keep the focus on texture–taste associations and influences.

## Methods

### Participants

A total of 529 participants were recruited for this study, 266 for the short questionnaire and 263 for the taste experiment. The short questionnaire was spread to a wide and varied sample via email, social media, personal contacts, and word of mouth. For the taste experiment, participants were recruited using a recruitment website that was spread on social media platforms, through friends, and through the family of the researchers involved in this project. Participants were required to have no taste impairment, be 16 years of age or older, be able to work with a computer, and be fluent in English. This procedure is consistent with the regulations of our Ethics Committee, who has approved the protocol of testing, privacy considerations, and COVID-safety precautions (G-2020-2671). The final sample for the short questionnaire data analysis consisted of 266 participants (*n = *266, age *M = *30.7, *SD = *11.8 years, 175 female, 89 male, one nonbinary, one preferred not to disclose). Eight participants who received the edible stimuli were excluded due to incorrect participation or failure to correctly enter the person-specific counterbalance code^1^ and 12 participants were excluded^2^ who presumably scanned the same experiment QR code without each completing the preceding sign-up procedure individually, resulting in a final sample of 247 for the taste experiment (*n = *247, age *M = *34.8, *SD = *14.8 years, 147 female). Participants did not receive reimbursement for participation.

### Power Analyses

Required sample sizes guided the attained number of participants, calculated by means of *a priori* power analyses based on similar studies. For the online questionnaire, we conducted an *a priori* power analysis using the pwr package in R developed by Stéphane Champley, based on Cohen (1977). We based ourselves on a study by Woods and Spence (2016), in which participants were instructed to assign color patches to one of the four taste words they were presented with. Our power analysis yielded a required sample size of 159 to achieve a power of .80. Based on this result, we aimed to reach between 160 and 200 participants.

For the taste experiment, we conducted two *a priori* power analyses, based on a study by Carvalho et al. (2017a). In this study, the influence of color on the experience of beer was investigated. Our first analysis, based on the results of a 2 × 2 repeated measures ANOVA, yielded a required sample size of 151 to achieve a power of .80. The second power analysis was based on the results of a Pearson's chi-square goodness-of-fit test. The chi-squared power calculation yielded a required sample size of 212 to achieve a power of .80. Based on these two power calculations, we aimed to recruit between 200 and 250 participants. The code is available in our OSF project under the name “PowerAnalyses” in the folder “Code + Data.”

### Study Design and Procedure

**Figure 1.**
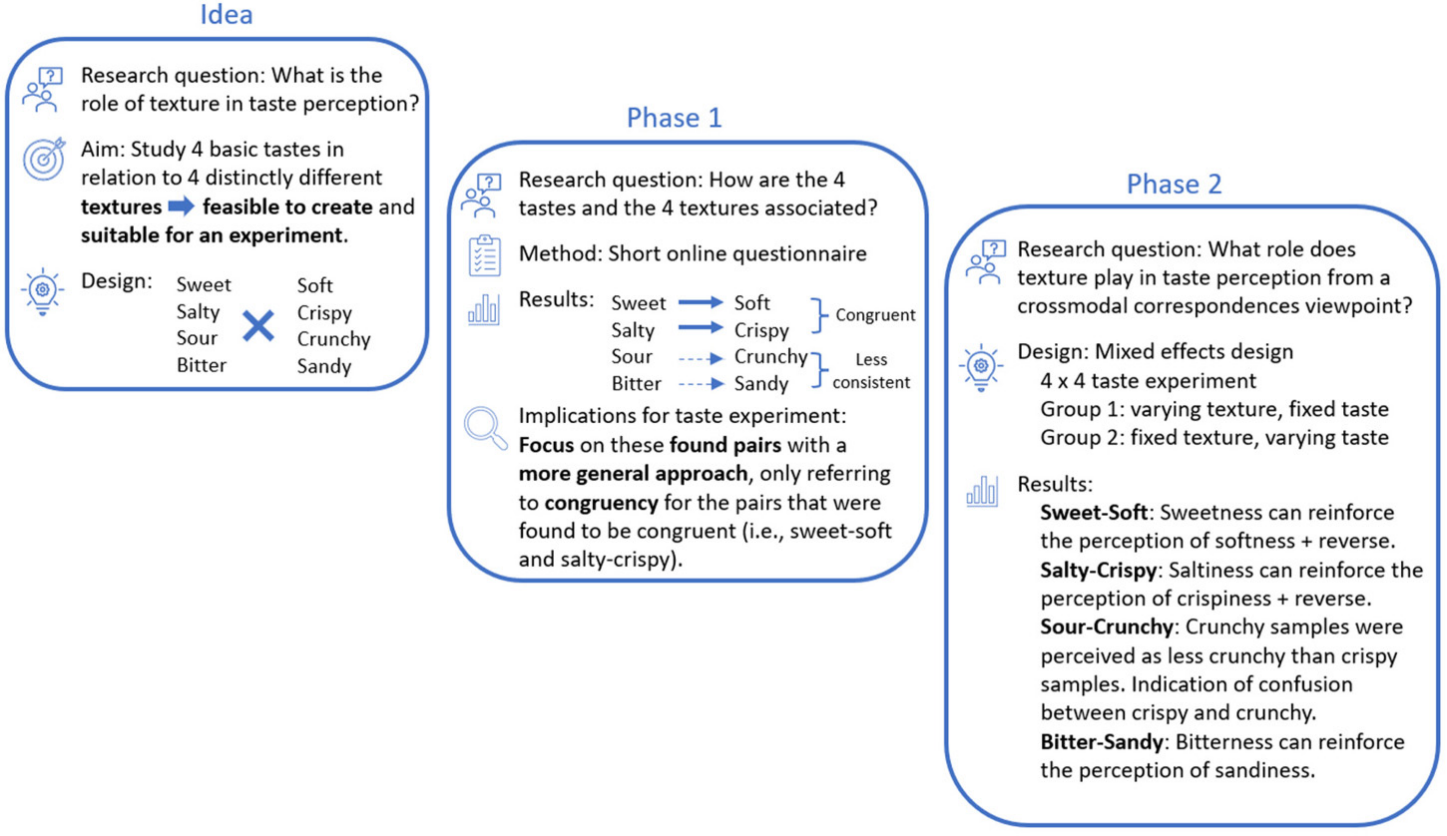
Advance organizer of each study phase, including research questions, methods, results and implications for subsequent phases.

In Figure 1, we provide an overview of the study phases, research questions, methods, results, and their implications for subsequent phases.

#### Short Questionnaire

The short online questionnaire (designed using Qualtrics, Provo, UT) started with the informed consent procedure. The first question (matching task) asked the participant to match different basic taste words (sweetness, sourness, bitterness, and saltiness) to different texture words (soft, crispy, crunchy, sandy) in a mutually exclusive design, with randomization of the order across participants. The texture terms were not defined to avoid the use of rules of thumb based on provided definitions, given that we aimed to learn how texture–taste associations are made spontaneously, that is, without steering participants in certain directions. The next question (prototype question) asked what specific food they associate with the different basic tastes. The last questions assessed age, gender, country of nationality, country of residence, and time abroad. The questionnaire allowed participants to add a second country of nationality and up to 10 countries of residence. Participants were debriefed by email after the finalization of the study, when they had indicated their wish to be debriefed during the informed consent procedure.

#### Taste Experiment

Participants of the taste experiment were recruited through a website that was shared on social media and with acquaintances of the researchers. The first step of signing up consisted of completing a form, in which informed consent was given, food allergies were assessed, demographics were asked, and safety measures with regard to COVID-19 were disclosed.

**Figure 2.**
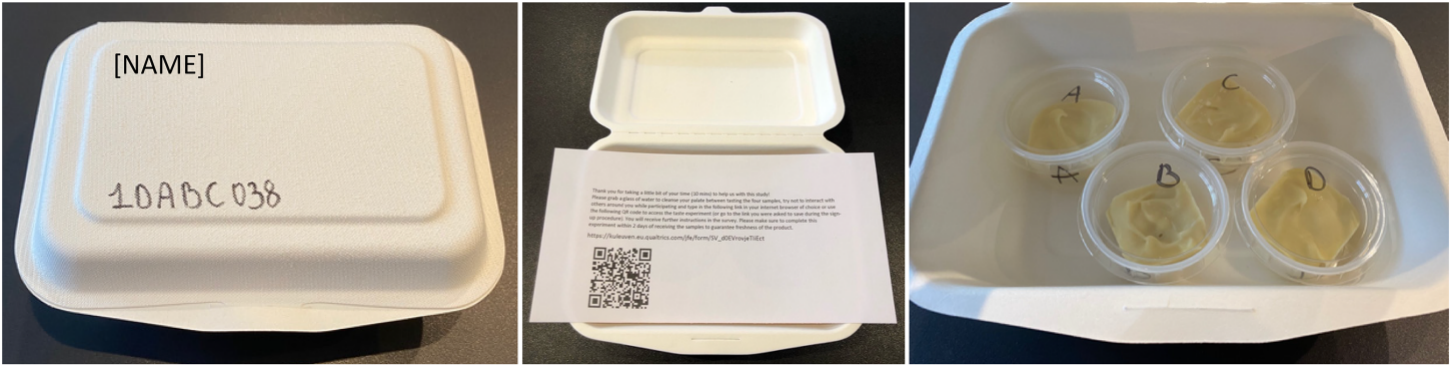
Packaging of the lunch boxes containing the stimuli and instruction.

Participants received a lunch box at home containing four samples labeled A, B, C, and D (see Figure 2).^3^ On the top of the box, a person-specific code and the names of the participants were written. As participants signed up, they were assigned to one of two groups (Group 1 with a fixed taste and varying textures, Group 2 with a fixed texture and varying tastes) in a four-by-four study design (4 tastes × 4 textures). In Group 1, 30 participants tasted sweet samples, 28 participants tasted salty samples, 31 participants tasted sour samples, and 29 participants tasted bitter samples. In Group 2, 32 participants tasted soft samples, 37 participants tasted crispy samples, 30 participants tasted crunchy samples, and 30 participants tasted sandy samples. The different tastes, textures, and the order in which they were to be consumed were counterbalanced across participants. The person-specific code on the top of the box was used to transmit information concerning which specific samples each participant was instructed to taste and the order (letters referred to the different textures, digits referred to the different tastes). Due to the counterbalancing taking place behind the scenes and the consistent use of the A, B, C, and D labels in alphabetic order, participants were unaware of the link between the code on top of the box and the specific samples they received.^4^ Inside the box, a small paper could be found with written explanations, instructing the participant to grab a glass of water to cleanse their taste palate between tasting the samples, to refrain from interacting with others while participating, and to access the taste experiment (online questionnaire with detailed instructions) using either the QR code on the paper or by typing the link (on the paper) in their internet browser.

Upon accessing the online questionnaire, participants were asked to enter the person-specific code. For each sample, participants were instructed to chew three times and then to start answering the questions right away. Both groups received the same questions. The first questions assessed texture perception (“To what extent do you perceive the following textures in this sample?”), texture liking (“How would you rate the sensation in your mouth when chewing this sample? Focus on the feeling in the mouth, not the taste.”), taste perception (“To what extent do you perceive the following tastes in this sample?”), and taste liking (“How would you rate the taste of this sample?”). In addition, participants were asked a question assessing the bodily sensation (“What kind of feeling does biting into this sample evoke within you?”) and were given a varied list of feeling-describing-words to pick from. These questions were repeated for each of the four stimuli. Further, four questions (prototype questions) assessing what specific food they associate with each basic taste were asked. Finally, the participants were instructed to match different basic tastes (sweetness, sourness, bitterness, and saltiness) to different textures (soft, crispy, crunchy, sandy) in a mutually exclusive design (matching task). Participants were informed that they would be debriefed afterward by email if they had indicated their wish to be debriefed during the informed consent procedure.

#### Stimuli

**Figure 3.**
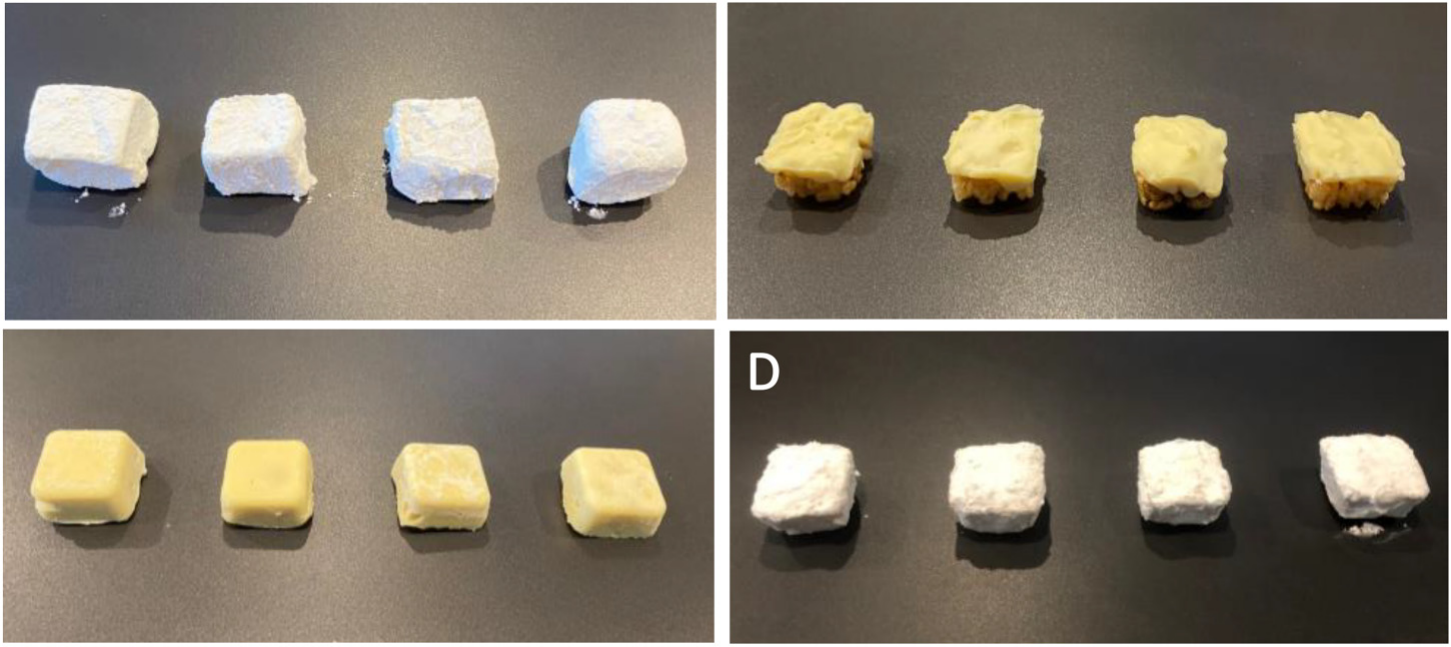
Sweet, salty, bitter, and sour stimuli in the textures soft (A), crispy (B), crunchy (C), and sandy (D).

The 16 stimuli for the taste experiment have been created with three goals in mind. Our first goal was to create the four different textures we are interested in studying, namely soft, crunchy, crispy, and sandy. The choice for these specific textures was driven by their obvious importance in everyday food textures (especially of snacks like sweets, cookies, potato chips, etc.), feasibility of creation, and suitability for an experiment. Our second goal was to vary the taste of each texture using the basic tastes of interest, namely sweetness, saltiness, sourness, and bitterness. Finally, considering the results of a previous study in which color has been found to exert a moderating effect on perceived texture (Chylinski et al., 2015), we aimed to create stimuli that differed minimally in color. All stimuli were white or cream-colored (see Figure 3).^5^ The volume of each stimulus was approximately 12.5 cm^3^. To standardize the mastication process over all stimuli, the soft samples were created to be slightly larger because, during consumption, these reduce in volume more quickly due to the soft, easily soluble base. The concentration of tastants was determined for each texture separately. After multiple rounds of experimenting and tasting, during which the concentrations of all tastants were adjusted, carefully weighed, and documented, we came to the compositions of ingredients that rendered for each texture a distinct taste, in all four basic tastes, without resulting in an aversive reaction. The avoidance of aversive reactions was crucial in this process. To maximize ecological validity, we aimed to create samples that do not carry an artificial character, meaning our goal was to use food that we encounter in everyday life. On these grounds, we decided that “sweet” was the most suitable baseline taste for each texture, given that it is a taste that is often combined with other tastes (e.g., in pralines, candy). The recipes for the samples are specified in Appendix A.

### Outcome Measures

#### Short Questionnaire

The short questionnaire consists of the following outcome measures.

**Texture and taste word associations.** The first task of the questionnaire instructed participants to match texture words with basic taste words in a mutually exclusive design. The different texture words and basic taste words were presented visually, meaning that participants were not able to freely produce words. The participant was not able to proceed to the next question until each basic taste word was matched with one texture word; therefore, there are no missing data for this outcome measure (forced-choice procedure).

**Prototype question.** Participants were asked to think of one specific food they associate with each basic taste (“What specific food do you associate with [sweetness]? Name one specific food (not a dish) that you find to be prototypical for [sweetness]”). Participants were able to freely name foods; incomprehensible words (because of extensive typos or use of dialects or slang) were transformed into missing data for analysis.

**Demographics.** This outcome measure is subdivided into “age,” “nationality,” “country of residence,” and “time abroad.” Participants were asked what their age is, what their country of nationality is, and were given the option to add a potential second country of nationality. They were additionally asked in what country they currently reside (most of the time), if they have lived in another country, in which country and for how long. The questionnaire allowed participants to add up to 10 countries of residence.

#### Taste Experiment

Preceding the experiment, demographics were assessed as described for the short questionnaire. The taste experiment itself comprises the following outcome measures.

**Texture rating.** First, participants were asked to rate the extent to which they perceive each texture in the sample. This rating was measured on an 11-point scale: “To what extent do you perceive the following textures in this sample?”: 0 = not at all, 10 = very much.

**Texture liking rating.** Then, liking of the texture was assessed on an 11-point scale: “How would you rate the sensation in your mouth when chewing this sample? Focus on the feeling in the mouth, not the taste.” 0 = very unpleasant, 10 = very pleasant.

**Taste rating.** The perceived taste was assessed in the same way as the perceived texture, namely by asking participants to rate the extent to which they perceived different tastes in the sample. This rating was measured on an 11-point scale: “To what extent do you perceive the following tastes in this sample?”: 0 = not at all, 10 = very much.

**Taste liking rating.** Similar to the second question, liking of the taste was assessed: “How would you rate the taste of this sample?”: 0 = very bad, 10 = very good.

**Sensation description.** The last question assessed the feeling that is evoked by chewing the samples. Participants were asked “What kind of feeling does biting into this sample evoke within you?” and were given a varied list of feeling-describing-words to pick from. This list comprised “pleasant,” “harmonic,” “uneasy,” “nails-on-chalkboard,” “neutral,” “nice,” “happy,” “disgust,” “strange,” “incongruent,” and “congruent.” They were instructed to indicate as many as they found applicable.

Finally, participants were asked the previously described prototype question and texture and taste associations matching question.

All materials (i.e., informed consent forms, short questionnaire, experiment preceding form, taste experiment questionnaire, and paper instructions that accompanied the box containing the samples) can be found in our OSF project under the folder “Supplementary Documents Manuscript.” Due to the complexity of some analyses, we will explicate each analysis before presenting the results of that analysis. All data analyses were conducted using the statistical program R (R Core Team, 2020) and the following packages: rcompanion (Mangiafico, 2016), ggplot2 (Wickham, 2016), weights (Pasek, 2013), and brms (Bürkner, 2017).

## Results

### Short Questionnaire

#### How Are Texture and Taste Words Matched?

Bonferroni-corrected Pearson chi-square tests of independence were performed for each texture word to examine how texture words and taste words were matched intuitively (see Table 1). The chi-square tests (*df *= 3, *N = *266) were significant at the alpha = .05 level for each texture, *X^2 ^*= 232.74, *p *< .0001, *V = *.54 for soft, *X^2 ^*= 13.16, *p *= .02, *V = *.13 for crunchy, *X^2 ^*= 151.23, *p *< .0001, *V = *.44 for crispy and *X^2 ^*= 66.18, *p *< .0001, *V = *.29 for sandy, suggesting there is a relation between the matching of taste words and texture words. Cramer's *V* is a measure of association between two variables and renders a value between 0 and 1.

**Table 1.** Frequencies of matches between texture words and taste words for the matching task data.

		Taste			
		Bitter	Salty	Sour	Sweet
Texture	Soft	49	4	43	170
	Crunchy	64	74	84	44
	Crispy	39	150	58	19
	Sandy	114	38	81	33

**Figure B.1. fig9-20416695231163473:**
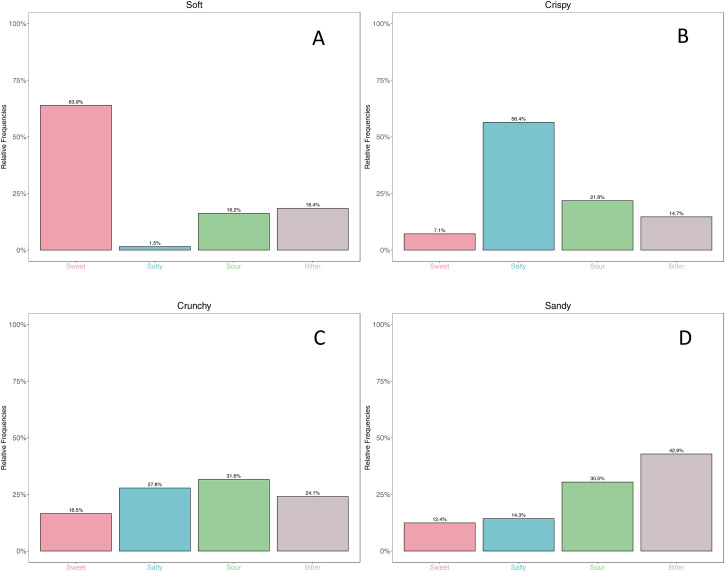
Relative frequencies of the matching of the tastes (sweet, salty, sour, and bitter) with the textures soft (A), crispy (B), crunchy (C), and sandy (D) in the matching task.

Post hoc Bonferroni pairwise comparisons for each texture word aimed to further investigate how each taste pair differed. The resulting tables and figures can be found in Appendix B. Table B.1 and Figure B.1(A) depict how soft was significantly paired with sweet, by 63.9% of all participants. Table B.2 and Figure B.1(B) indicate significant pairing of crispy with salty, by 56.4% of all participants. Table B.3 and Figure B.1(C) show a less consistent pairing concerning the crunchy texture, with both sour and salty matched to crunchy above chance level (25%). Table B.4 and Figure B.1(D) indicate a less consistent pattern of pairing concerning the sandy texture, with both bitter and sour having been assigned to sandy by 42.9% and 30.5% of all participants, respectively.

#### Do Cross-Cultural Differences Exist in how Textures and Tastes Are Matched?

Cross-cultural differences in texture and taste word associations were analyzed by means of weighted chi-square tests of independence^6^ between culture and the matching of texture and taste words for each texture. The culture data consisted of countries that were later assigned to regional cuisines, relying on a list provided by the United Nations (UN, 2018). Each country was assigned a weight according to three criteria. Participants with multiple nationalities were given equal weights for each reported nationality and each reported country of residence; only countries in which participants resided for over a year were recorded as a cultural influence. The chi-square tests showed nonsignificant results, suggesting no association between culture and texture–taste word associations. However, given the large group of West Europeans, we are reluctant to draw strong conclusions from this analysis. More details are available in the OSF project.

#### Do the Textures of the Prototypical Food Products for Each Taste Correspond with how Textures and Tastes Were Paired in the Matching Task?

The prototype question assessed what specific food products participants found to be prototypical for each taste. Each food product was assigned a texture label following an inter-rater consensus procedure. This procedure was applied by one of the researchers and two volunteers. The first part consisted of a pilot coding round based on 10% of the data, followed by a discussion during which the raters refined the criteria. This refined coding scheme was then applied by each rater individually on the remaining data. For the final coding, the majority rule was applied. The resulting texture labels were used to conduct Bonferroni-corrected chi-square tests of independence between texture and taste, for each taste (see Table 2).

**Table 2.** Frequencies of matches between texture and taste for the prototype data.

		Texture			
		Soft	Crunchy	Crispy	Sandy
Taste	Bitter	145	114	0	6
	Salty	81	12	166	6
	Sour	232	31	2	0
	Sweet	169	74	0	23

The Bonferroni-corrected Pearson chi-square tests of independence, performed to compare to the results of the chi-square tests of the matching task data, yielded significant results for all tastes, *X*^2^(*df *= 2, *N *= 266) = 123.84, *p *< .0001, *V = *.48 for sweet, *X^2^*(*df *= 3, *N *= 265) = 252.69, *p *< .0001, *V = *.56 for salty, *X^2^*(*df *= 2, *N *= 265) = 355.25, *p *< .0001, *V = *.82 for sour and *X^2^*(*df *= 2, *N *= 265) = 120.55, *p *< .0001, *V = *.48 for bitter, indicating there is a relation between the texture of the assigned food products and the taste.

Post hoc Bonferroni pairwise comparisons were calculated for each taste to further investigate how the different textures of the entered food products differed in relation to one another for each taste. The resulting tables and figures can be found in Appendix B.

**Figure B.2. fig10-20416695231163473:**
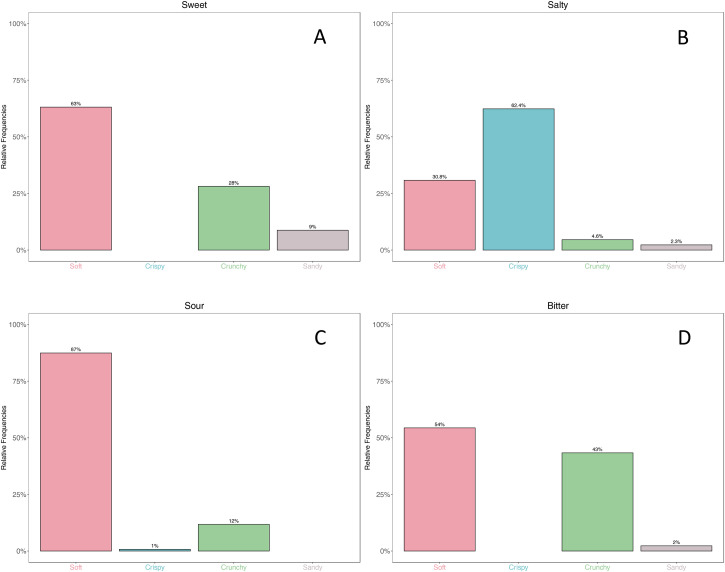
Relative frequencies of the tastes sweet (A), salty (B), sour (C), and bitter (D) and the textures (soft, crispy, crunchy, and sandy) of the food products entered in the prototype question.

Table B.5 and Figure B.2(A) indicate the significant assignment of food products with a soft texture to the taste sweet, by 63% of all participants. Table B.6 and Figure B.2(B) indicate that 62.4% of all participants assigned a food product with a crispy texture to the taste salty and 30.8% assigned a food product with a soft texture to the taste salty. Table B.7 and Figure B.2(C) indicate significant assignment of a food product with a soft texture to the sour taste, by 87% of all participants. Table B.8 and Figure B.2(D) show the significant assignment of soft and crunchy foods to the taste bitter, by 54% and 43% of all participants, respectively.

The percentages presented in Table 3 show the 10% most common responses of food products per taste, including the texture.

**Table 3.** Responses reported by more than 10% of all participants.

Food product per taste	Texture	Percentage
Sweet		
Candy	Soft	24.44%
Chocolate	Crunchy	21.43%
Salty		
Chips	Crispy	50.00%
Sour		
Lemon	Soft	60.90%
Candy	Soft	12.03%
Bitter		
Grapefruit	Soft	21.43%
Chicory	Crunchy	19.55%

### Taste Experiment

The first two research questions allow us to compare the results of the participants in the taste experiment with the results of the short questionnaire. For these analyses, we did not discriminate between the two groups of participants (with varying textures or tastes).

#### How are Textures and Tastes Matched?

The Bonferroni-corrected Pearson chi-square tests of independence applied to the data from the matching task, completed by participants of the taste experiment (*df *= 3, *N *= 259), suggested there is an association between the matching of textures with tastes (see Table 4). Specifically, the tests yielded *X^2 ^*= 196.43, *p *< .0001, *V = *.50 for the soft texture, *X^2 ^*= 31.27, *p *< .0001, *V = *.20 for the crunchy texture, *X^2 ^*= 113.74, *p *< .0001, *V = *.38 for the crispy texture and *X^2 ^*= 32.69, *p *< .0001, *V = *.21 for the sandy texture.^7^

**Table 4.** Frequencies of matches between texture and taste for the matching task data.

		Taste			
		Bitter	Salty	Sour	Sweet
Texture	Soft	26	11	66	156
	Crunchy	88	51	85	35
	Crispy	43	138	49	29
	Sandy	102	59	59	39

**Figure C.1. fig11-20416695231163473:**
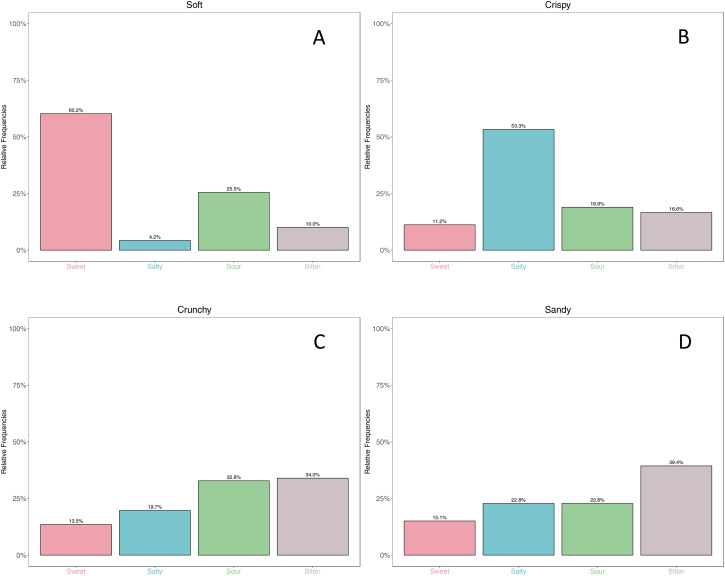
Relative frequencies of the matching of the tastes (sweet, salty, sour, and bitter) with the textures soft (A), crispy (B), crunchy (C), and sandy (D) in the matching task.

Post hoc Bonferroni pairwise comparisons were calculated for a closer inspection of differences between pairs of tastes within each texture. The resulting tables and figures can be found in Appendix C. Table C.1 and Figure C.1(A) indicate that soft was matched with sweet and sour by 60.2% and 25.5% of all participants, respectively. Table C.2 and Figure C.1(B) indicate the largest significant pairing of crispy with salty, by 53.3% of all participants. Table C.3 and Figure C.1(C) rendered notably smaller differences with bitter and sour having been paired with crunchy by 34.0% and 32.8% of all participants, respectively. Table C.4 and Figure C.1(D) show a significant pairing of bitter with sandy, by 39.4% of all participants.

#### Do the Textures of the Prototypical Food Products for Each Taste Correspond with how Textures and Tastes Were Paired in the Matching Task?

The prototype data was investigated in the same way as for the short questionnaire (see Table 5). These tests yielded significant results for all tastes, specifically *X^2^ (df *= 3, *N *= 258) = 192.42, *p *< .0001, *V = *.50 for sweet, *X^2^*(*df *= 3, *N *= 257) = 115.09, *p *< .0001, *V = *.39 for salty, *X^2^*(*df *= 2, *N *= 257) = 397.83, *p *< .0001, *V = *.88 for sour, and *X^2^*(*df *= 3, *N *= 258) = 239.83, *p *< .0001, *V = *.56 for bitter.

**Table 5.** Frequencies of matches between texture and taste for the prototype data.

		Texture			
		Soft	Crunchy	Crispy	Sandy
Taste	Bitter	139	113	2	4
	Salty	107	28	107	15
	Sour	236	20	0	1
	Sweet	146	84	2	26

**Figure C.2. fig12-20416695231163473:**
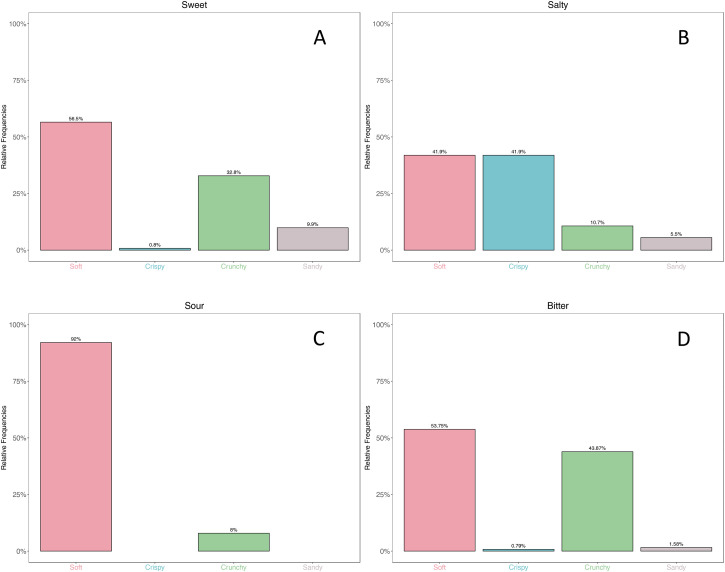
Relative frequencies of the tastes sweet (A), salty (B), sour (C), and bitter (D) and the textures (soft, crispy, crunchy, and sandy) of the food products entered in the prototype question.

Post hoc Bonferroni pairwise texture comparisons were applied to offer a closer look at the differences among the textures in the matching of textures to each taste.^8^ The resulting tables and figures can be found in Appendix C. Table C.5 and Figure C.2(A) indicate that both soft and crunchy foods were assigned to sweet above chance level, by 56.5% and 32.8% of all participants, respectively. Table C.6 and Figure C.2(B) show that for the salty taste, food products with crispy and soft texture were primarily entered, both by 41.9% of all participants. Table C.7 and Figure C.2(C) indicate that 92% of all participants were assigned a food product with soft texture to sour. Table C.8 and Figure C.2(D) indicate that foods of soft and crunchy texture were assigned to bitter by 53.75% and 43.87% of all participants, respectively.

 Table 6 presents the 10% most common responses of food products per taste, including the texture.

**Table 6.** Responses reported by more than 10% of all participants.

Food product per taste	Texture	Percentage
Sweet		
Chocolate	Crunchy	27.80%
Candy	Soft	23.17%
Salty		
Chips	Crispy	29.73%
Anchovies	Soft	12.36%
Sour		
Lemon	Soft	65.25%
Candy	Soft	14.29%
Bitter		
Chicory	Crunchy	28.96%
Grapefruit	Soft	25.48%
Coffee	Soft	10.43%

### Multilevel Ordinal Logistic Regression Analysis

**Figure 4.**
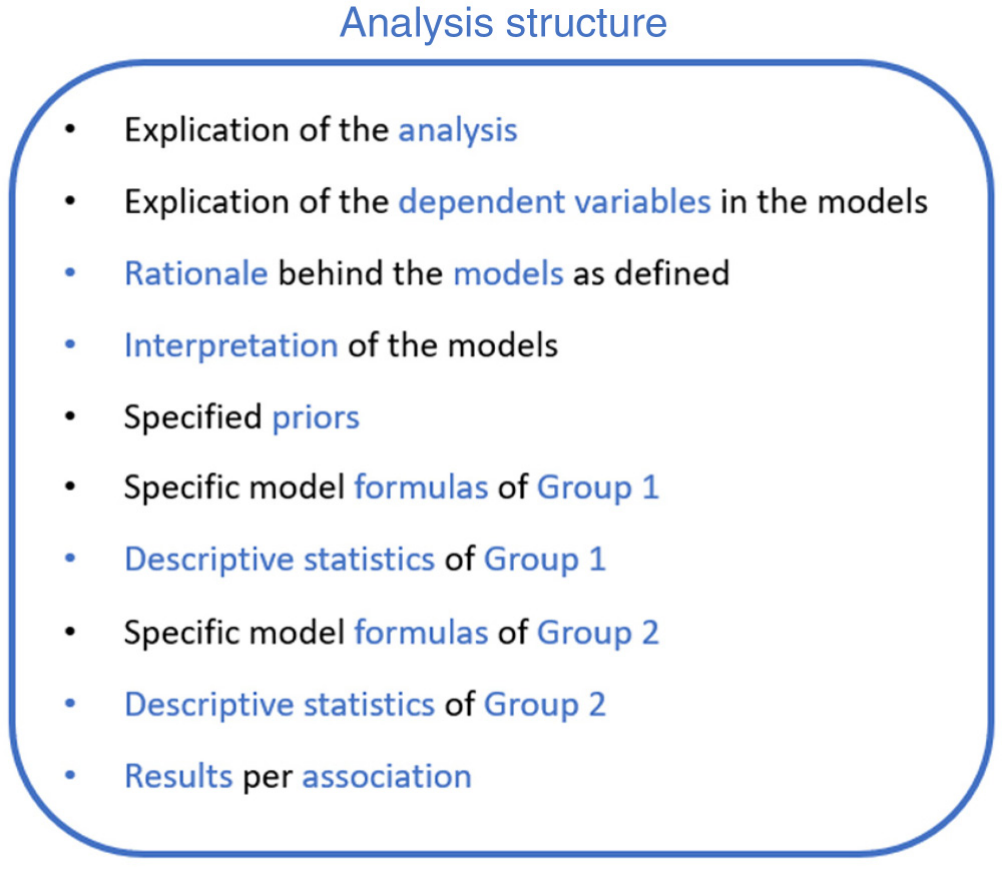
Advance organizer of multilevel ordinal logistic regression analysis.

The taste experiment data were analyzed using Bayesian^9^ mixed effects models by means of Stan computational framework (Stan Development Team, 2020) (see advance organizer Figure 4). Group 1 (varying texture, fixed taste) and Group 2 (varying taste, fixed texture) were analyzed separately with 10 multilevel ordinal logistic regression models for each group. For both groups, four models were fitted for the dependent variable “ExtentTexture” (one for each investigated texture), one model was fitted for the dependent variable “SensationRating,” four models were fitted for the dependent variable “ExtentTaste” (one for each investigated taste) and one model was fitted for the dependent variable “TasteRating.” The reasoning behind this extensive investigation of each dependent variable according to the investigated predictor level lies in the explorative character of the study. This regression analysis was approached in a more general manner than we had initially planned. We intended to create congruent pairs depending on the results of the short questionnaire; however, given that the data of the short questionnaire did not render consistent congruent pairs for each texture and taste, we decided on a more general approach with a focus on the most consistent associations (i.e., sweet-soft, salty-crispy, sour-crunchy, and bitter-sandy), only referring to congruency when appropriate (i.e., for sweet-soft and salty-crispy). Potential effects of (in)congruency were investigated by assigning each model to reference levels that corresponded to the matches between taste and texture found in the short questionnaire, allowing the comparison with other taste and texture pairings to be made. More specifically, for the models that investigated soft texture, sweet and soft were applied as reference levels and for the models that investigated crispy texture, salty and crispy were applied as reference levels. Although the matching of crunchy and sandy was less consistent, the models that investigated crunchy texture were estimated with sour and crunchy as reference levels, and the models that investigated sandy texture were estimated with bitter and sandy as reference levels. In such a manner, the results of each regression model show whether there are significant differences between the textures or the tastes in perceived taste, perceived texture, etc. For instance, a model with “soft” and “sweet” as reference levels and “ExtentTexture” as the dependent variable can indicate whether sweet-soft samples were perceived as more soft than bitter-soft samples.

We specified mildly informative priors to improve convergence and guard against overfitting.^10^ In this paper, we will only present the most relevant findings related to our research questions, focusing on the most consistently associated pairs rendered by the results of the short questionnaire, from a crossmodal correspondence perspective. On these grounds, we focus on one taste in relation to one texture for each found pair. A complete overview of all 20 multilevel ordinal logistic regression models^11^ can be found in our OSF project under the folder “Supplementary Documents Manuscript,” with inclusion of convergence checks, posterior predictive checks, and complete results.

Group 1 (varying texture, fixed taste)

For Group 1, the model formulas included fixed effects for “Taste,” “Texture” and its interaction, and a random effect for the intercept and for “Taste,” nested in “Subject.”

[Dependent Variable] ∼ 1 + Taste + Texture + Taste:Texture + (1 + Taste | Subject)

**Figure 5.**
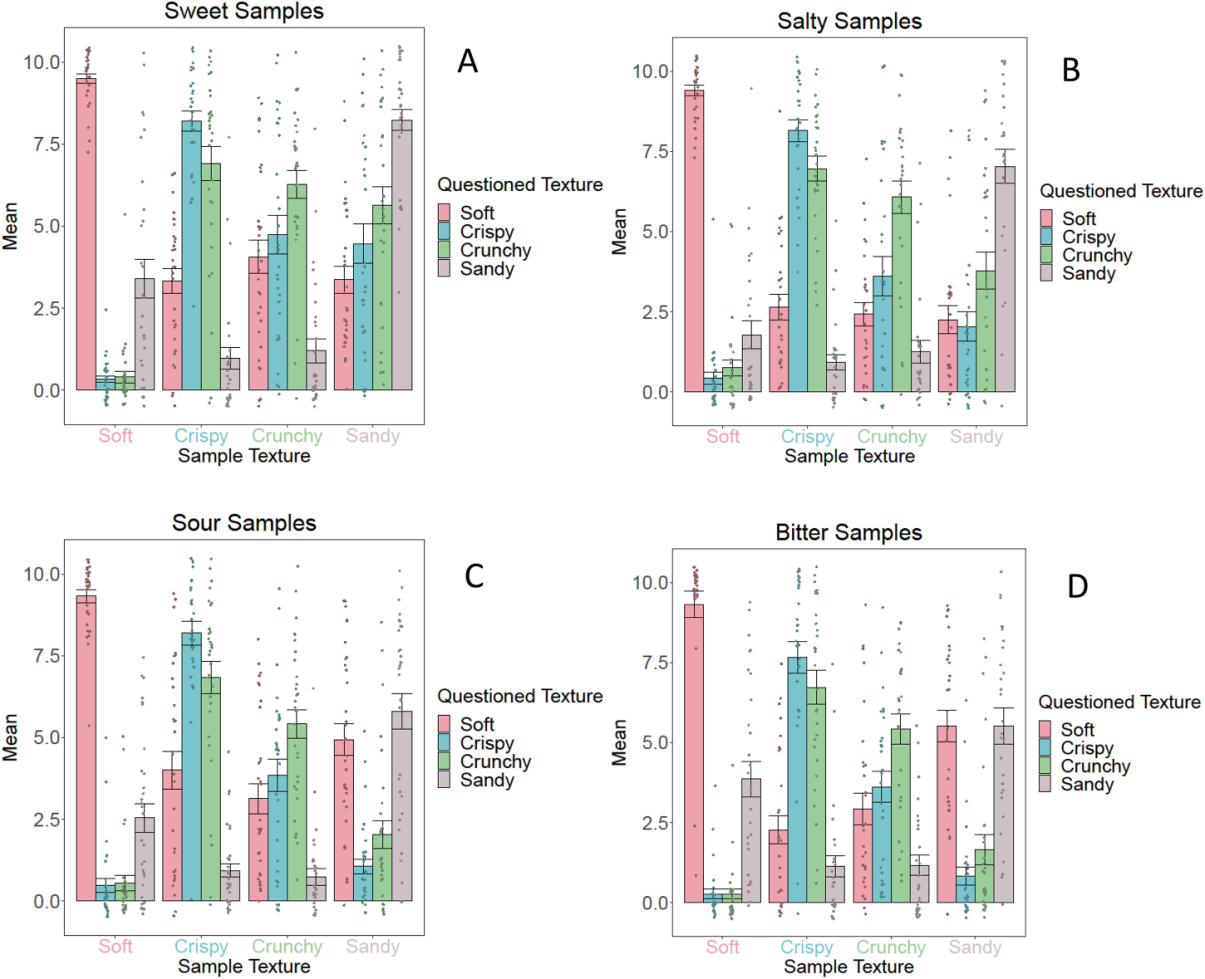
Mean *texture* ratings including standard errors for the sweet samples (A), salty samples (B), sour samples (C), and bitter samples (D) for *Group 1* (fixed taste, varying texture). These graphs illustrate the descriptive statistics of each sample. The legend describes the dependent variable (i.e., the assessed texture), the x-axis shows the texture of the samples.

**Figure 6.**
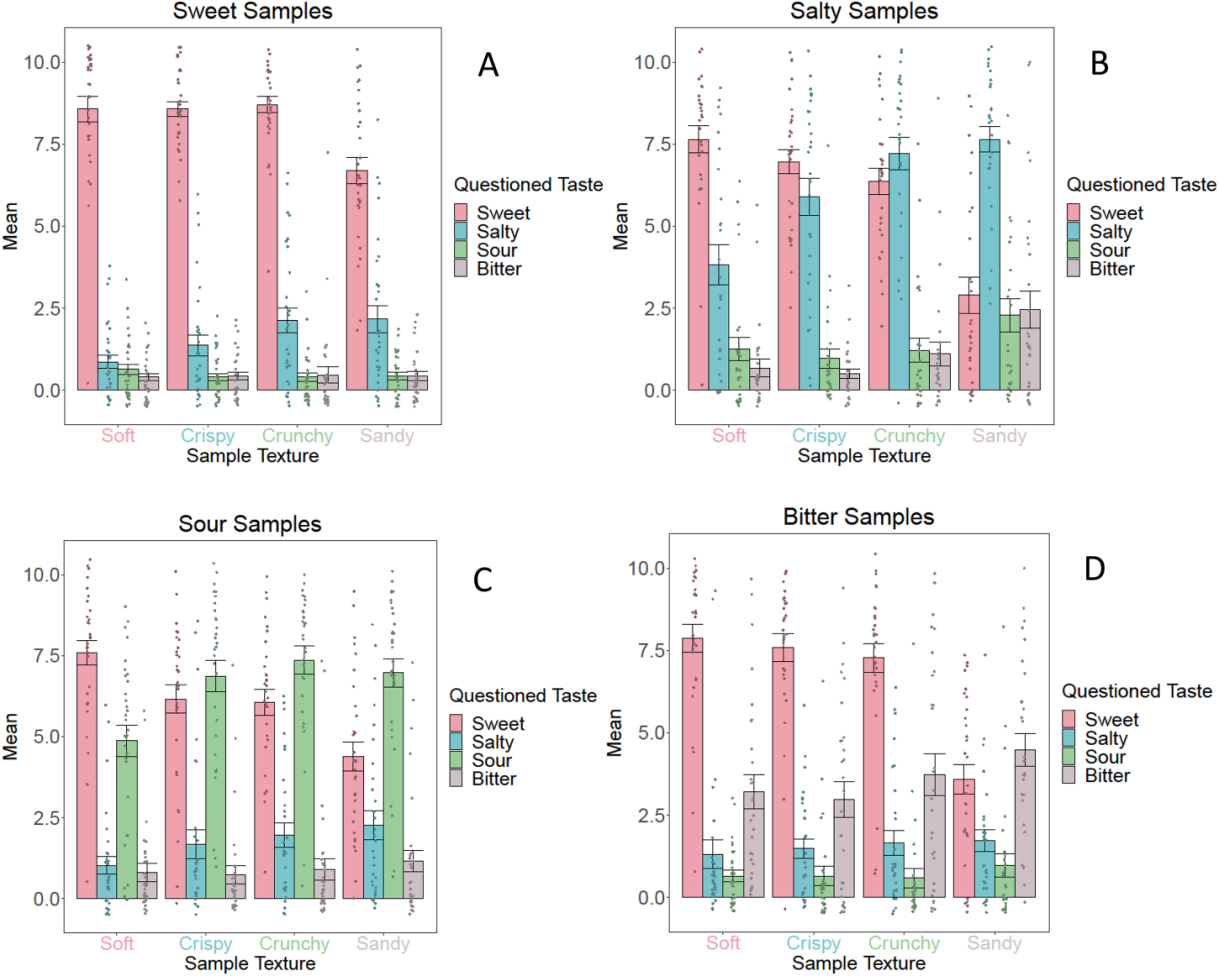
Mean *taste* ratings including standard errors for the sweet samples (A), salty samples (B), sour samples (C), and bitter samples (D) for *Group 1* (fixed taste, varying texture). These graphs illustrate the descriptive statistics of each sample. The legend describes the dependent variable (i.e., the assessed taste), and the *x*-axis shows the texture of the samples.

 Figures 5 and 6 visualize the descriptive statistics of Group 1 for the texture ratings and the taste ratings, respectively.

Concerning the crunchy samples, the extent to which they were rated crunchy compared to the crispy samples is noteworthy. As can be seen in Figure 5, although crunchy samples were rated highest on crunchy compared to other possible textures, the crispy samples were rated to be crunchy to an even higher extent than the crunchy samples.

Group 2 (varying taste, fixed texture)

For Group 2, the model formulas included fixed effects for “Texture,” “Taste” and its interaction, and a random effect for the intercept and for “Texture,” nested in “Subject.”

[Dependent Variable] ∼ 1 + Texture + Taste + Texture:Taste + (1 + Texture | Subject)

**Figure 7.**
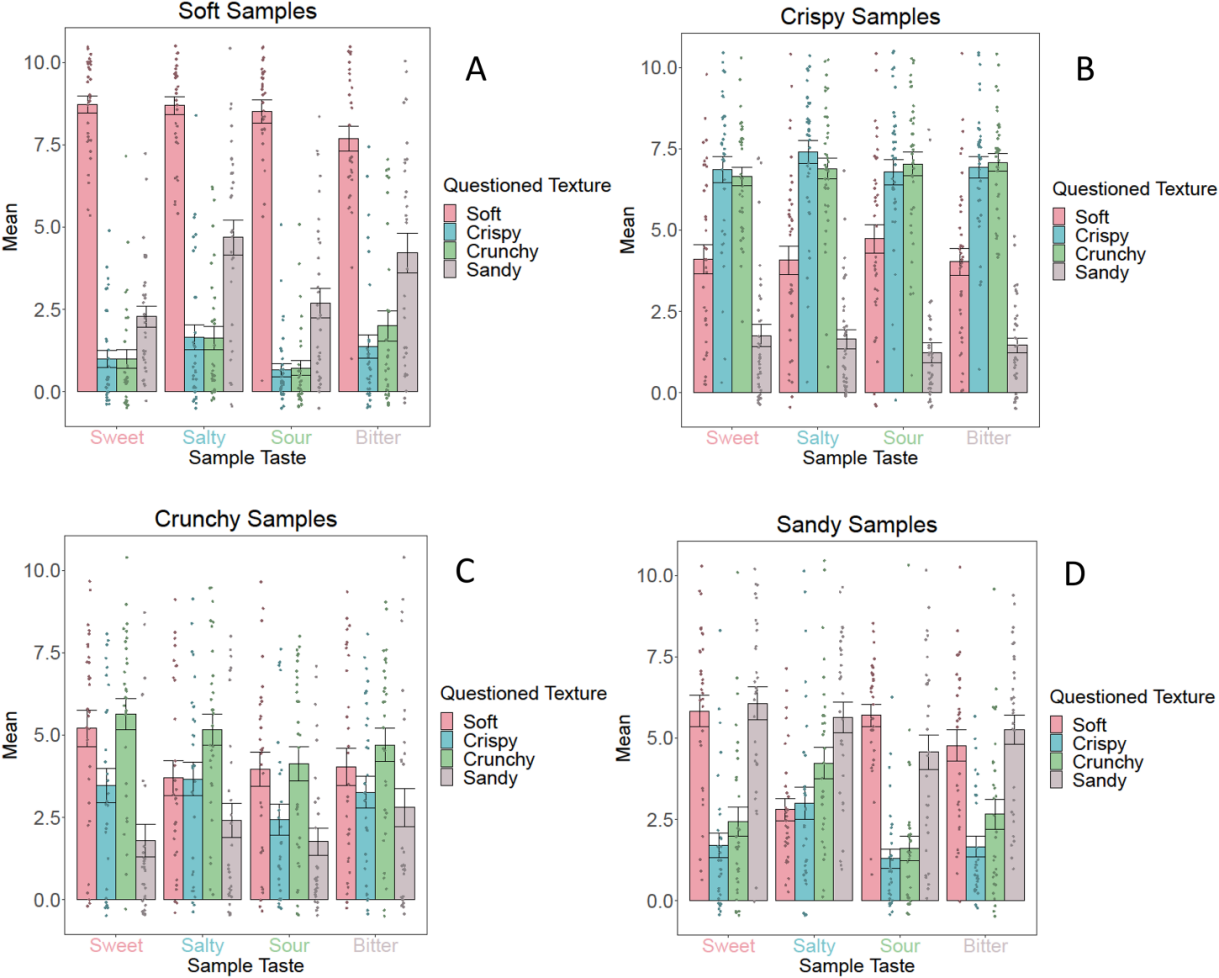
Mean *texture* ratings including standard errors for the soft samples (A), crispy samples (B), crunchy samples (C), and sandy samples (D) for *Group 2* (fixed texture, varying taste). These graphs illustrate the descriptive statistics of each sample. The legend describes the dependent variable (i.e., the assessed texture), and the *x*-axis shows the taste of the samples.

**Figure 8.**
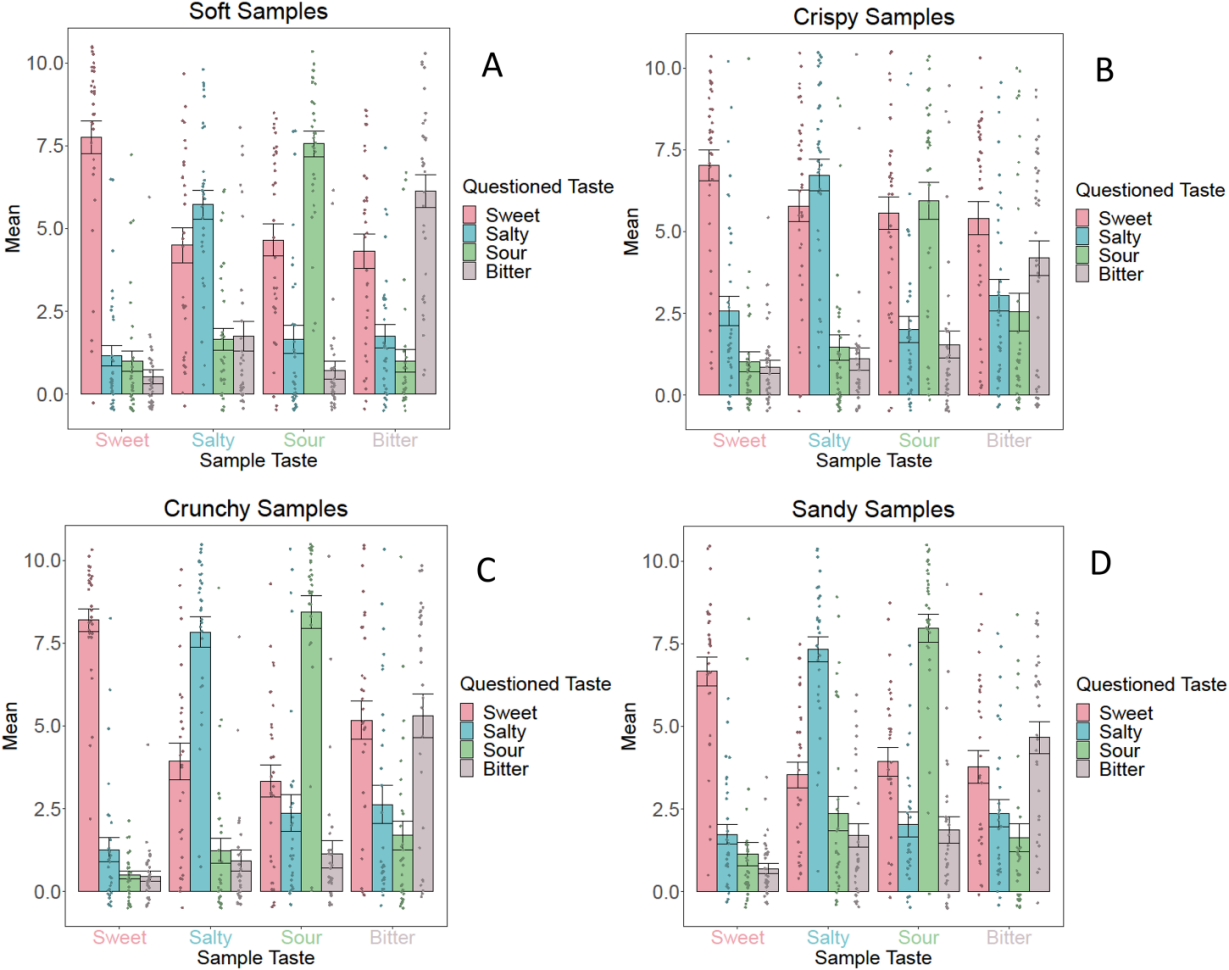
Mean *taste* ratings including standard errors for the soft samples (A), crispy samples (B), crunchy samples (C), and sandy samples (D) for *Group 2* (fixed texture, varying taste). These graphs illustrate the descriptive statistics of each sample. The legend describes the dependent variable (i.e., the assessed taste), and the *x*-axis shows the taste of the samples.

 Figures 7 and 8 visualize the descriptive statistics of Group 2 for the texture ratings and taste ratings, respectively.

#### Sweet and Soft Association

**Figure C.3. fig13-20416695231163473:**
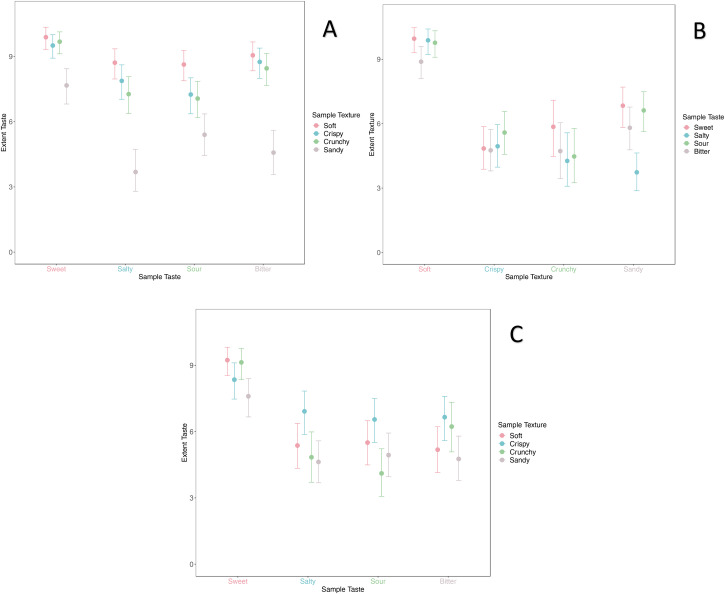
Conditional effects of model Sweet1 (A), model Soft2 (B), and model Sweet2 (C) visualizing the mean extent to which each taste or texture was perceived given the sample taste and texture with standard error of the mean.

Model “Sweet1” yielded for “TextureSandy” a point estimate of −2.58 with 95% CI [−3.6, −1.56], indicating that for Group 1, sweet-soft samples were perceived to be more sweet than sweet-sandy samples, as can be observed in Figure C.3(A).

The model “Soft2” yielded for “TasteBitter” a point estimate of −1.66 with 95% CI [−2.72, −.63], indicating that sweet-soft samples were perceived to be more soft than bitter-soft samples. This result can be observed in Figure C.3(B).

The model “Sweet2” yielded for “TextureSandy” the point estimate −1.55 with 95% CI [−2.59, −.54], indicating that sweet-soft samples were perceived to be more sweet than the sweet-sandy samples, as can be observed in Figure C.3(C).

#### Salty and Crispy Association

**Figure C.4. fig14-20416695231163473:**
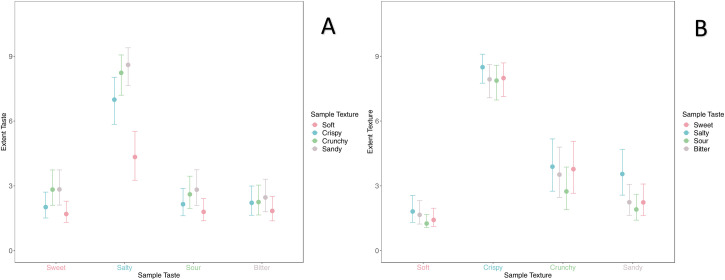
Conditional effects of model Salty1 (A) and model Crispy2 (B) visualizing the mean extent to which each taste or texture was perceived given the sample taste and texture with standard error of the mean.

The model “Salty1” yielded for “TextureSoft” a point estimate of −2.06 with 95% CI [−3.12, −1.00], indicating that salty-crispy samples were perceived as more salty than salty-soft samples, illustrated by Figure C.4(A). The interactions “TasteSour:TextureSoft,” with a point estimate of 1.49 and 95% CI [.03, 2.95], and “TasteSweet:TextureSoft,” with a point estimate of 1.50 and 95% CI [.06, 2.96], regarding the extent to which the taste salty was perceived, suggest that the difference between crispy and soft was larger when taste was salty than when taste was sour or sweet.

The model “Crispy2” yielded for “TasteSour” a point estimate of −.87 with 95% CI [−1.73, −0.03], indicating that salty-crispy samples were perceived to be more crispy than sour-crispy samples, as can be observed in Figure C.4(B). The interaction “TextureSandy:TasteSour” with a point estimate of −1.39 and 95% CI [−2.74, −0.07] indicates that the difference between salty and sour was larger for the texture sandy than for the texture crispy.

#### Sour and Crunchy Association

**Figure C.5. fig15-20416695231163473:**
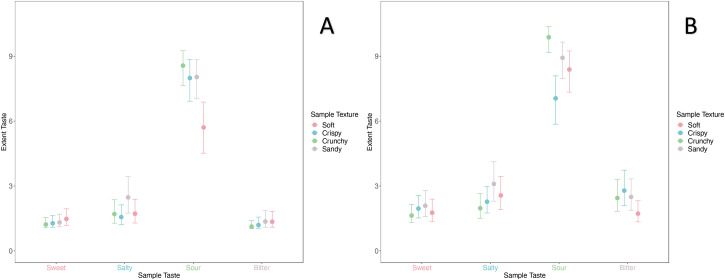
Conditional effects of model Sour 1 (A) and model Sour 2 (B) visualizing the mean extent to which each taste was perceived given the sample taste and texture with standard error of the mean.

The model “Sour1” yielded for “TextureSoft” a point estimate of −2.42 with 95% CI [−3.42, −1.42], indicating that sour-crunchy samples were perceived to be more sour than sour-soft samples. This result is illustrated by Figure C.5(A).

The model “Sour2” rendered for “TextureCrispy” a point estimate of −2.36 and 95% CI [−3.44, −1.3] and for “TextureSoft” a point estimate of −1.46 and 95% CI [−2.53, −.38], indicating that sour-crunchy samples were perceived to be more sour than sour-crispy samples and sour-soft samples, as can be observed in Figure C.5(B). The interaction “TextureCrispy:TasteBitter” with a point estimate of 2.69 and 95% CI [1.32, 4.07] indicates that concerning the extent to which the taste sour was perceived, there was a larger difference between the sour and bitter-crunchy samples than between the sour and bitter-crispy samples.

#### Bitter and Sandy Association

**Figure C.6. fig16-20416695231163473:**
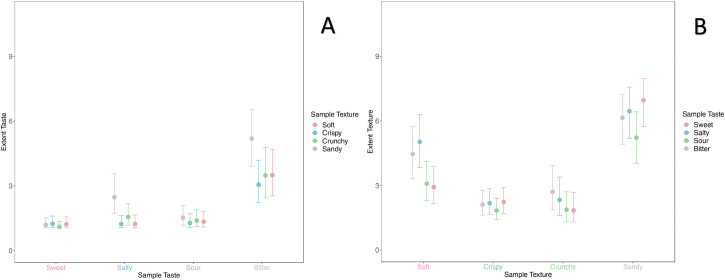
Conditional effects of model Bitter 1 (A) and model Sandy 2 (B) visualizing the mean extent to which each taste or texture was perceived given the sample taste and texture with standard error of the mean.

The model “Bitter1” yielded for “TextureCrispy” a point estimate of −1.85 and 95% CI [−2.87, −.85], for “TextureCrunchy” a point estimate of −1.41 and 95% CI [−2.47, −.37] and for “TextureSoft” a point estimate of −1.41 and 95% CI [−2.38, −.43], indicating that bitter-sandy samples were perceived as more bitter than bitter-crispy, crunchy, and soft samples. These results are illustrated in Figure C.6(A).

The model “Sandy2,” yielded for the interaction “TextureSoft:TasteSweet” a point estimate of −2.25 with 95% CI [−3.58, −.94], conveying that bitter-soft samples were perceived to be more sandy than sweet-soft samples, whereas bitter-sandy samples were perceived to be less sandy than sweet-sandy samples. These results can be observed in Figure C.6(B).

## Discussion and Conclusion

### Short Questionnaire

The matching task of the short questionnaire yielded less consistent associations for crunchy and crispy. Given that salty was consistently paired with crispy, and crunchy with both salty and sour, above chance level, may imply that participants confused crispy and crunchy. This confusion around the distinction between “crisp” and “crunchy” does not come as a surprise as this has previously been discussed in various studies (Spence, 2015; Varela & Fiszman, 2012; Varela et al., 2007).

The results of the prototype data suggested that participants did not rely on an availability heuristic when instructed to match the texture words to taste words intuitively. As such, the textures of the entered food products did not yield the same pairings as those found in the matching task. For the salty taste, which was consistently paired with the crispy texture during the matching task, the recorded food products comprised crispy textures but also soft textures in 30.8% of the cases. The discrepancy in this pairing of textures with tastes between the matching task and the prototype question suggests that the relation between texture and taste emerges from an intuitive feeling rather than from experience, or active and easily retrievable examples. This sort of intuitive association has previously been described by Köhler (1947), who found that the rounded sounding word “maluma” was more often associated with organic (round and smooth) shapes, whereas the harsher sounding word “takete” was more often associated with angular shapes.

This is further supported by the fact that the results of the cross-cultural analysis yielded no significant relation between culture and pairing of textures with tastes. Taking into consideration that international restaurants have sprung up like mushrooms in the last 50 years, facilitating the globalization of cuisines, it is not surprising that only a few cultural food habits have maintained their unknown or alien character in our highly connected world. However, as mentioned previously, we are reluctant to draw strong conclusions from this cross-cultural analysis due to the large group of West Europeans in the sample.

### Taste Experiment

The matching task conducted by the participants in the taste experiment largely confirmed the results found in the independently conducted short questionnaire in another sample. For the crunchy texture, once again, no consistent pairing was found. However, in this sample, participants paired crunchy with bitter and sour above chance level. Compared to the matching of crunchy with both sour and salty for the short questionnaire, the difference between crispy and crunchy may have led to confusion, potentially resulting in crunchy also being paired with salty above chance level. The experience of the taste experiment may have led to a more refined distinction between crispy and crunchy after participation, potentially explaining why participants of the taste experiment did not pair crunchy with salty above chance level (the matching task was presented after the samples were consumed). The prototype data analysis of the taste experiment sample largely confirmed the results for the sample of the short questionnaire. The salty taste, which was found to be paired consistently with crispy during the matching task in both samples, was assigned with food products of both soft and crispy textures with equal proportions in the prototype question. This finding provides further support that experience does not seem to be of particular importance in the relation between texture and taste.

### Sweet and Soft Association

The intuitive association between sweet and soft, which emerged consistently in the short questionnaire, was also reflected in the results of the taste experiment. For both groups, sweet-soft samples were perceived to be more sweet than sweet-sandy samples. In addition, a reinforcing effect of the sweet taste on the soft texture was found, considering sweet-soft samples were perceived as softer than bitter-soft samples. This effect of congruency is in line with the evidence found for the pairing of sweet with soft in the short questionnaire and in previous research (Spence & Ngo, 2012) and calls attention to the potential of sweetness to evoke or invigorate the perception of softness as well as the reverse.

### Sour and Crunchy Assocation

Concerning the crunchy samples, the results of the taste experiment reveal that the crunchy samples were perceived as more crunchy than the other investigated textures, while the crunchy samples were also perceived as less crunchy than the crispy samples. This could have resulted from the possible confusion between crunchy and crispy we previously alluded to. The complexity concerning the crunchy texture may arise from two sources of confusion. First, the findings of the short questionnaire pointed toward a more complex association between taste and crunchy texture. Specifically, there seems to be uncertainty around the semantic distinction between crispy and crunchy, given that crunchy was matched with salty above chance level in the short questionnaire, potentially indicating confusion between potato chips being crunchy or crispy, for instance. The question arises as to where this confusion stems from. Besides the potential linguistic problem due to different languages using different terms or having no terms to describe certain textures (Spence, 2015), marketing in the food industry oftentimes employs mouthfeel-describing words such as crunchy and crispy to render a heightened appeal to a product. In advertisements for chips, the word crunchy is often used (Cheetos, 1948). Alternatively, crunchy is also used in advertisements for chocolate-nut bars such as Snickers® Cruncher (May, 2005). Although experience does not seem to lie at the basis of how we associate textures and tastes, the marketing seems to stick and may result in confusion when we are asked to pair a certain taste with a texture such as crunchy, which is used to make not only chips but also chocolate-nut bars more appealing. This alternating between labeling dry foods as not only crispy but also crunchy could be a deliberate effort to capture the consumer's attention through a mismatch, considering that Johnson and Pfenninger (2021) have found evidence suggesting incongruent context, applied through labels, resulted in significantly higher ratings of taste than the congruent context. This mismatch has been suggested to render increased attention to the sensory experience (Donohue et al., 2013). Given that attention has been proposed as an important factor in food choice (Bialkova et al., 2013), this deliberate element of surprise may serve as a tactic to encourage the selection of a certain bag of potato chips among the plethora of options one gets to choose from nowadays. Halkias and Kokkinaki (2013) have previously presented the effectiveness of applying incongruity-based tactics in advertising.

Aside from the influence advertisements might have on our daily lives and the confusion such inconsistencies may bring about, there may be a second aspect that contributes to the complexity surrounding the crunchy texture and taste association. Phonetics may be at play in the perceived crunchiness of the crispy samples, particularly for the taste experiment in this case. The crunchy sound, emerging when consuming crispy samples, may evoke the perception of crunchiness more than the crunchy samples due to the airiness of the crispy samples, allowing the crunchy sound to travel more easily through the eustachian tube. This may lead to a more profound echo during consumption. This is in line with previous research for which a “creamy” soundtrack seemed to have an enhancing effect on the perceived creaminess and sweetness of chocolate (Carvalho et al., 2017b). Although the reinforcing effect found for sour-crunchy samples being perceived as more sour than sour-soft samples indicates support toward the association found between sour and crunchy in the short questionnaire, the concern pertaining to the reliability of the crunchy samples discourages us to draw conclusions regarding this relation. The apparent confusion between crunchy and crispy that came to light in this study will have to be investigated more closely by means of a taste experiment design in which various types and levels of crunchy and crispy textures are considered.

### Salty and Crispy Association

Regarding the crispy samples, our findings provide support for the previously found congruent match between crispy and salty. Salty-crispy samples were found to be perceived as more crispy than sour-crispy samples. Additionally, salty-crispy samples were perceived as more salty than salty-soft samples. Like the sweet-soft association, this interaction suggests that saltiness has the potential to render the sensation of other textures as more crispy, perhaps as a result of its intuitive association with saltiness.

### Bitter and Sandy Association

The analysis of the sandy texture partially shows support for the association between sandy and bitter and alternatively demonstrates the complexity regarding this relation, previously also found in the short questionnaire. In line with the association between bitter and sandy, alluded to by the results of the short questionnaire, bitter-sandy samples were perceived to be more bitter than bitter-crispy, bitter-crunchy, and bitter-soft samples. Alternatively, bitter-soft samples were perceived as more sandy than sweet-soft samples, whereas bitter-sandy samples were perceived to be less sandy than sweet-sandy samples. It is noteworthy, however, that the interaction indicated a larger difference between bitter-soft and sweet-soft than between bitter-sandy and sweet-sandy. In addition, the 95% credible intervals of the bitter-sandy and sweet-sandy samples showed more overlap. This result mainly suggests that the bitter taste has the potential to evoke the perception of sandy in soft samples, although as the findings of the short questionnaire suggested, the association between sandy and bitter is more complex and irreducible to a simple intuitive match.

The liking data we have collected suggested large individual differences with no clear tendencies. Because of the multitude of data, we have decided to only discuss the relevant findings of this study in this paper. All the analyses of the liking and sensation data can be found in our OSF project.

### Limitations of the Current Study

One limitation of the current study we have previously mentioned is the visual appearance of the stimuli. Although we aimed to equate the visual appearance of the stimuli as much as possible, upon close inspection, the stimuli still contain subtle visual cues that might be suggestive of their texture qualities. In particular, when considering the visual appearance of the samples (see Figure 3), two confounding variables may hamper the pure tactile perception of the textures by chewing (i.e., the mouthfeel). The texture samples fall apart in two groups by color: A (soft) and D (sandy) are more white, while B (crispy) and C (crunchy) are more yellowish (“creamy”). Moreover, they fall apart in two other groups by visually perceived smoothness-roughness as follows (for a review, see Fleming, 2017): A (soft) and C (crunchy) are more smooth, while B (crispy) and D (sandy) are more rough. These two visual dimensions may have influenced the texture and taste perception in the group of participants who received the samples with varying textures (Group 1). Because the four separate textures as intended in our study are confounded by these two visual dimensions in entangled pairs, it is impossible to disentangle the tactile effects from the visual effects. However, several of our findings indicate that this confounding has probably played a minimal role, if any. Importantly, the results do not differ much between the two versions of the experiment (i.e., Group 1 with varying textures and Group 2 with fixed textures), which suggests that the within-group visual appearance of the stimuli (i.e., which varies in Group 1 and does not vary in Group 2) cannot have had a big effect. Next, examining the texture ratings for the four different tastes in Group 1 (Figure 5), not even one of the 16 comparisons shows signs of a bipartite division along the visual smoothness-roughness dimension (textures A–C = smooth vs. B–D = rough). Seven of the 16 show a sign of a bipartite division along the color dimension (textures A–D = white vs. B–C = yellow) but in each of these the textures within a pair (either A–D or B–C) also differ, suggesting that more than color is at play. Furthermore, examining the texture–taste associations by multilevel ordinal logistic regression analysis, the models never indicate a simple bipartite division along the color dimension (white vs. yellow) or the roughness-smoothness dimension (see Figures C.3 to C.6). As an example, for the model “Sweet 1” (Figure C.3A), the perceived taste for the sandy sample is always lower than the three other samples. Therefore, we are confident that our results are not exclusively due to the visual appearance of our texture samples but of course we cannot completely rule out that they have somehow interacted with the pure mouthfeel effects. This also implies that we recommend future studies to try to better equate the visual appearance of food samples when focusing on tactile texture and taste (which is quite challenging when aiming for ecologically valid stimuli) or to blindfold their participants (which may have other disadvantages).

A second limitation of this study was the absence of an experimenter during participation in the taste experiment, which implies that no control was possible over whether instructions were followed properly. However, the exceptional circumstances in which we gathered the data (i.e., a pandemic) meant that we had to make do with the means available, meaning relying on participants to follow the instructions correctly. If anything, this reduced control would have dampened rather than strengthened the effects reported here.

A third limitation of this study is the limited number of textures explored. Because our study primarily aimed to investigate whether consistent associations are made between taste and texture, we chose a limited number of textures for the purpose of feasibility. In addition, each texture, as we specified it, had only one specific exemplar, so the findings may be specific to our particular operationalization. Future research should try to design similar samples with equal textures in order to try to replicate and generalize the present findings. Moreover, considering we now have a first grasp on taste and texture correspondences, it could be useful to explore a larger number of textures, which may further clarify the complexities found in this study.

### General Conclusion

The results of the current study show support for consistent associations between soft and sweet. These textures were paired with sweet and salty taste, respectively, in both the short questionnaire and the taste experiment. Concerning the textures crunchy and sandy, the current study has found evidence suggesting that the associations are more complex. In addition, we can conclude participants rely more on their intuition than on experience with familiar foods for this association. The taste experiment largely confirmed the conclusions we drew from the short questionnaire and in some cases offered a sense of clarity. More specifically, the taste experiment found evidence in favor of the consistent pairing of sweet with soft and salty with crispy, previously found in the short questionnaire. The less consistent pairing of sour with crunchy and bitter with sandy was also demonstrated by the taste experiment. Regarding the relation between sour and crunchy, the taste experiment revealed the complexity of studying crossmodal correspondences in taste perception. As such, there seemed to be confusion surrounding the difference between the texture terms crunchy and crispy. Overall, this study recognizes the substantial role of texture in taste perception and suggests that the associations between texture and taste arise intuitively, from feelings rather than from external factors such as experience. In conclusion, the findings of this study convey the relevance to further explore how textures can influence or modulate the perception of taste and in general, the gastronomic experience.

## Appendix A: Recipes Taste Experiment

### Soft

For the soft stimuli (Figure 2A), a marshmallow recipe was used to create small cubes of marshmallows, consisting of sugar, agave syrup, powdered gelatin, vanilla extract, salt, and water. The classic recipe makes sweet marshmallows. The agents we used to create sour, salty, and bitter marshmallow cubes are citric acid, salt, and coffee, respectively. These agents were added to the batter itself. Finally, the cubes were coated in a mixture of corn starch and powdered sugar^12^.

Concentrations per batch:

- Sugar: 410 g
- Agave syrup: 85 g
- Powdered gelatin: 21.6 g
- Vanilla extract: 1 tablespoon
- Salt: 1 teaspoon
- Water: 240 ml
- Taste agents: 5 g of each

### Crispy

The crispy stimuli (Figure 2B) were created based on Rice Krispies® treats. These bites consisted of Rice Krispies® cereal, simple syrup, the above-mentioned taste varying agents, and a thin layer of white chocolate as a topping. The same cube divided pan was used to prepare these stimuli to ensure a roughly similar shape as the crunchy stimuli.

Concentrations per batch:

- Simple syrup (1/2 sugar, ½ water): 10 tablespoons
- Rice Krispies® cereal: 90 g
- Taste agents: ¼ teaspoon of each per sample
- Thin layer of white chocolate on top: 1 teaspoon per sample

### Crunchy

The crunchy stimuli (Figure 2C) consisted of white chocolate and almonds. The same agents as described above were added to the center of the stimuli to vary the taste. These chocolate-nut cubes were made in a baking pan with a small cube division.

Concentrations per sample:

- 2/3 white chocolate
- 1/3 almonds
- Taste agents: ¼ teaspoon of each per sample

### Sandy

The sandy stimuli (Figure 2D) were small sand cookies consisting of almond flour, butter, sugar, all-purpose flour, vanilla extract, egg yolk, and again the taste varying agents. The cookies were also baked in the cube divided pan and lastly covered with powdered sugar.

Concentrations per batch:

- Almond flour: 1 cup
- Butter: 1 ½ cup
- Sugar: 1/2 cup
- All-purpose flour: 5 cups
- Vanilla extract: 1 tablespoon
- Egg yolk: 1

Taste agents: 5 g of each

## Appendix B: Short Questionnaire

[Fig fig9-20416695231163473] [Fig fig10-20416695231163473] [Table table7-20416695231163473] [Table table8-20416695231163473] [Table table9-20416695231163473] [Table table10-20416695231163473] [Table table11-20416695231163473] [Table table12-20416695231163473] [Table table13-20416695231163473] [Table table14-20416695231163473]

**Table B.1. table7-20416695231163473:** Pairwise taste comparisons for the soft texture of the matching task data*.*

Pairwise comparisons of chi-square tests for soft texture		
		*p*
Taste pairs	Bitter:Salty	3.82e−09
	Bitter:Sour	1.00e+00
	Bitter:Sweet	1.75e−15
	Salty:Sour	7.68e−08
	Salty:Sweet	1.54e−35
	Sour:Sweet	1.96e−17

**Table B.2. table8-20416695231163473:** Pairwise taste comparisons for the crispy texture of the matching task data.

Pairwise comparisons of chi-square tests for crispy texture		
		*p*
Taste pairs	Bitter:Salty	4.08e−15
	Bitter:Sour	3.22e−01
	Bitter:Sweet	5.18e−02
	Salty:Sour	1.07e−09
	Salty:Sweet	4.19e−23
	Sour:Sweet	5.29e−05

**Table B.3. table9-20416695231163473:** Pairwise taste comparisons for the crunchy texture of the matching task data.

Pairwise comparisons of chi-square tests for crunchy texture		
		*p*
Taste pairs	Bitter:Salty	1.00
	Bitter:Sour	0.60
	Bitter:Sweet	0.33
	Salty:Sour	1.00
	Salty:Sweet	0.05
	Sour:Sweet	0.002

**Table B.4. table10-20416695231163473:** Pairwise taste comparisons for the sandy texture of the matching task data.

Pairwise comparisons of chi-square tests for sandy texture		
		*p*
Taste pairs	Bitter:Salty	4.24e−09
	Bitter:Sour	1.09e−01
	Bitter:Sweet	1.43e−10
	Salty:Sour	4.85e−04
	Salty:Sweet	1.00e+00
	Sour:Sweet	4.16e−05

**Table B.5. table11-20416695231163473:** Pairwise texture comparisons for the sweet taste of the prototype data*.*

Pairwise comparisons of chi-square tests for sweet taste		
		*p*
Texture pairs	Crunchy:Sandy	6.72e−07
	Crunchy:Soft	8.64e−09
	Sandy:Soft	7.29e−25

**Table B.6. table12-20416695231163473:** Pairwise texture comparisons for the salty taste of the prototype data*.*

Pairwise comparisons of chi-square tests for salty taste		
		*p*
Texture pairs	Crispy:Crunchy	1.30e−29
	Crispy:Sandy	5.09e−33
	Crispy:Soft	6.84e−07
	Crunchy:Sandy	9.42e−01
	Crunchy:Soft	5.02e−12
	Sandy:Soft	5.35e−15

**Table B.7. table13-20416695231163473:** Pairwise texture comparisons for the sour taste of the prototype data*.*

Pairwise comparisons of chi-square tests for sour taste		
		*p*
Texture pairs	Crispy:Crunchy	1.34e−06
	Crispy:Soft	3.51e−50
	Crunchy:Soft	2.18e−34

**Table B.8. table14-20416695231163473:** Pairwise texture comparisons for the bitter taste of the prototype data*.*

Pairwise comparisons of chi-square tests for bitter taste		
		*p*
Texture pairs	Crunchy:Sandy	1.88e−22
	Crunchy:Soft	2.11e−01
	Sandy:Soft	9.39e−29

## Appendix C: Taste Experiment

[Fig fig11-20416695231163473] [Fig fig12-20416695231163473] [Fig fig13-20416695231163473] [Fig fig14-20416695231163473] [Fig fig15-20416695231163473] [Fig fig16-20416695231163473] [Table table15-20416695231163473] [Table table16-20416695231163473] [Table table17-20416695231163473] [Table table18-20416695231163473] [Table table19-20416695231163473] [Table table20-20416695231163473] [Table table21-20416695231163473] [Table table22-20416695231163473]

**Table C.1. table15-20416695231163473:** Pairwise taste comparisons for the soft texture of the matching task data*.*

Pairwise comparisons of chi-square tests for soft texture		
		*p*
Taste pairs	Bitter:Salty	8.22e−02
	Bitter:Sour	1.82e−04
	Bitter:Sweet	3.37e−21
	Salty:Sour	2.20e−09
	Salty:Sweet	1.94e−28
	Sour:Sweet	9.24e−09

**Table C.2. table16-20416695231163473:** Pairwise taste comparisons for the crispy texture of the matching task data*.*

Pairwise comparisons of chi-square tests for crispy texture		
		*p*
Taste pairs	Bitter:Salty	9.90e−12
	Bitter:Sour	1.00e+00
	Bitter:Sweet	5.94e−01
	Salty:Sour	4.56e−10
	Salty:Sweet	1.99e−16
	Sour:Sweet	1.41e−01

**Table C.3. table17-20416695231163473:** Pairwise taste comparisons for the crunchy texture of the matching task data*.*

Pairwise comparisons of chi-square tests for crunchy texture		
		*p*
Taste pairs	Bitter:Salty	1.02e−02
	Bitter:Sour	1.00e+00
	Bitter:Sweet	1.06e−05
	Salty:Sour	2.13e−02
	Salty:Sweet	5.07e−01
	Sour:Sweet	3.01e−05

**Table C.4. table18-20416695231163473:** Pairwise taste comparisons for the sandy texture of the matching task data*.*

Pairwise comparisons of chi-square tests for sandy texture		
		*p*
Taste pairs	Bitter:Salty	4.21e−03
	Bitter:Sour	4.21e−03
	Bitter:Sweet	6.72e−07
	Salty:Sour	1.00e+00
	Salty:Sweet	2.60e−01
	Sour:Sweet	2.60e−01

**Table C.5. table19-20416695231163473:** Pairwise texture comparisons for the sweet taste of the prototype data*.*

Pairwise comparisons of chi-square tests for sweet taste		
		*p*
Texture pairs	Crispy:Crunchy	9.30e−18
	Crispy:Sandy	5.75e−05
	Crispy:Soft	6.84e−31
	Crunchy:Sandy	1.43e−07
	Crunchy:Soft	3.95e−04
	Sandy:Soft	5.23e−19

**Table C.6. table20-20416695231163473:** Pairwise texture comparisons for the salty taste of the prototype data*.*

Pairwise comparisons of chi-square tests for salty taste		
		*p*
Texture pairs	Crispy:Crunchy	4.43e−11
	Crispy:Sandy	2.72e−16
	Crispy:Soft	1.00e+00
	Crunchy:Sandy	2.54e−01
	Crunchy:Soft	4.43e−11
	Sandy:Soft	2.72e−16

**Table C.7. table21-20416695231163473:** Pairwise texture comparisons for the sour taste of the prototype data*.*

Pairwise comparisons of chi-square tests for sour taste		
		*p*
Texture pair	Crunchy:Soft	6.81e–41

**Table C.8. table22-20416695231163473:** Pairwise texture comparisons for the bitter taste of the prototype data*.*

Pairwise comparisons of chi-square tests for bitter taste		
		*p*
Texture pairs	Crispy:Crunchy	6.84e−24
	Crispy:Sandy	1.00e+00
	Crispy:Soft	2.32e−29
	Crunchy:Sandy	1.15e−22
	Crunchy:Soft	6.72e−01
	Sandy:Soft	4.01e−28

## Notes

1. Participants were divided into two groups and received the different stimuli, labeled as A, B, C, and D, in a counterbalanced order. This code was also used to link the first part of the taste experiment (demographic questions) with the second part (the actual taste experiment) as explained further.

2. These participants were excluded because they may have shared the stimuli that were meant for one participant and therefore did not follow the instructions correctly.

3. Although the labels A, B, C, and D can be suggestive of order and therefore do not provide completely blind coding, the circumstances we were bound by (i.e., COVID restrictions resulting in at-home experiments) forced us to depend on participants’ cooperation in adherence with the elaborate instructions we provided. In this trade-off between blind coding and clear instructions, we believe that the correct order of consumption takes precedence over completely blind coding, given that incorrect order of consumption would jeopardize the results of this experiment. The results should be robust to labelling effects given the counterbalancing of the order, in which each label was assigned to all 16 different samples while keeping the labels and their order of consumption consistent across all participants (i.e., the randomization took place behind the scenes and all participants were instructed to taste samples A, B, C, and D in that order).

4. Unless they discussed the specifics of their experience with other participants, in which case the link could have been made only after participation.

5. An important limitation that has to be taken into consideration for the interpretation of the results of this study is the fact that the stimuli do not look absolutely identical, although we have tried our best to reduce any visible differences. The different textures posed a challenge in creating stimuli of identical appearance. The idea to coat all stimuli using a fixed component has been considered; however, we believe this would compromise the perception of texture.

6. Although the preregistration described that cross-cultural differences would be investigated using Spearman correlations, upon inspection of the data, it became clear that weighted Chi-square tests of independence were more suitable, taking the categorical nature of the data into consideration. This raw weight method was applied because in the case of multiple responses per participant, the classical Pearson Chi-square test’s assumption of independence is violated (Decay & Thomas, 2000).

7. The sample consisted of 259 participants for this analysis. For analyses that concern the actual taste experiment, responses of 247 participants are investigated because this number of participants could be relied on to have consumed the samples correctly (e.g., not having shared the samples with others, considering this violates the instructions to consume the samples correctly for the purpose of the experiment). Additionally, for cross-cultural analyses 247 participants are investigated because others could not be linked correctly to the demographic questions assessed during sign-up.

8. For the pairwise comparisons, seven participants were removed due to missing responses because the “pairwisenominalindependence” R-function required a complete matrix without missing values. Consequently, some textures were not tested in a pairwise manner despite containing a value larger than zero in Table 5 (e.g., sandy concerning the taste sour).

9. We opted for a Bayesian analysis in this part because a frequentist model estimation could be problematic because of the large number of factors included in the model. In addition, the intuitive interpretation of Bayesian statistics is another advantage when dealing with a complex analysis.

10. The choice for the distribution of the models was guided by the recommendation not to treat rating data as metric (Liddell & Kruschke, 2018). We decided to apply the cumulative family with a logit link because it allowed the model to generate samples that approximated the data of our experiment sample more accurately. In order to apply the cumulative(logit) family to the model, the data, initially ranging from 0-10, was rescaled to 1-11.

11. Upon inspection of the actual data and given the exploratory nature of this study, we decided to analyze the data using 20 multilevel ordinal logistic regression models as opposed to the mixed model ANOVAs we described in the preregistration.

12. In the preregistration (included in our OSF project), we described that the taste varying agents would be added to the coating, however due to the coffee resulting in a slight darkening of the samples, we have decided before the start of the study to limit the addition of all taste agents to only the batter. This also applies to the sandy samples.
